# Enhancing LoRaWAN Performance Using Boosting Machine Learning Algorithms Under Environmental Variations

**DOI:** 10.3390/s25134101

**Published:** 2025-06-30

**Authors:** Maram A. Alkhayyal, Almetwally M. Mostafa

**Affiliations:** Department of Information Systems, College of Computers and Information Sciences, King Saud University, Riyadh 11451, Saudi Arabia; almetwaly@ksu.edu.sa

**Keywords:** boosting algorithms, environmental variations, LoRaWAN, path loss

## Abstract

Accurate path loss prediction is essential for optimizing Long-Range Wide-Area Network (LoRaWAN) performance. Previous studies have employed various Machine Learning (ML) models for path loss prediction. However, environmental factors such as temperature, humidity, barometric pressure, and particulate matter have been largely neglected. This study bridges this gap by evaluating the performance of five boosting ML models—AdaBoost, XGBoost, LightGBM, GentleBoost, and LogitBoost—under dynamic environmental conditions. The models were compared with theoretical models (Log-Distance and Okumura-Hata) and existing studies that employed the same dataset based on metrics such as RMSE, MAE, and R^2^. Furthermore, a detailed performance vs. complexity analysis was conducted using metrics such as training time, inference latency, model size, and energy consumption. Notably, barometric pressure emerged as the most influential environmental factor affecting path loss across all models. Bayesian Optimization was applied to fine-tune hyperparameters to improve model accuracy. Results showed that LightGBM outperformed other models with the lowest RMSE of 0.5166 and the highest R^2^ of 0.7151. LightGBM also offered the best trade-off between accuracy and computational efficiency. The findings show that boosting algorithms, particularly LightGBM, are highly effective for path loss prediction in LoRaWANs.

## 1. Introduction

The exponential growth of the Internet of Things (IoT) is transforming both urban and rural ecosystems through an interconnected web of devices. These devices are often deployed in remote, inaccessible, or resource-constrained environments that require communication systems that prioritize long-range coverage, energy efficiency, and cost-effectiveness. This need has led to the rise of Low-Power Wide-Area Networks (LPWANs), among which the LoRaWAN (Long-Range Wide-Area Network) has emerged as a dominant protocol [1]. The LoRaWAN has the ability to support battery-powered devices with lifetimes of several years. It also supports the capacity for wide-area communication over the unlicensed spectrum, which makes it especially suitable for several fields and applications, such as industry, healthcare, and environmental monitoring [2].

These IoT applications present several operational constraints. Devices that are known as End Nodes (ENs), are often deployed in the contexts of forests, mountains, or wildlife to address the sector-specific challenges [3]. In the agricultural domain, the LoRaWAN has demonstrated its potential through deployments in palm oil plantations in Malaysia, where traditional wireless systems struggled with signal degradation caused by dense foliage and terrain [4]. In such settings, devices must communicate reliably while minimizing power consumption, as replacing batteries may be impractical or impossible [5]. The LoRaWAN addresses this challenge through key configurable parameters like Spreading Factor (SF), Transmission Power (TP), and Bandwidth (BW), which determine the trade-off between data rate, range, and energy use. However, choosing the optimal configuration is highly sensitive to environmental dynamics and network density [6]. Among other use cases, smart city initiatives have also adopted the LoRaWAN to power applications across diverse urban and indoor settings. In large geographical outdoors, extensive sensor networks using the LoRaWAN have enabled the real-time monitoring of parameters such as CO2 levels [7], temperature, humidity, and motion [8].

Despite its advantages, the performance of the LoRaWAN can be significantly influenced by various environmental conditions and deployment-specific characteristics. A major challenge in LoRaWAN planning lies in accurately predicting Path Loss (PL), which quantifies signal attenuation during transmission [9]. Traditional PL models, such as Log-Distance or Okumura-Hata, typically rely on geometric parameters like distance and frequency [1]. It has been observed that several environmental dynamics such as temperature, humidity, and barometric pressure are usually ignored as they all can substantially alter signal propagation. Therefore, there is a need to incorporate real-world variability into PL estimation methods. In addition, the Adaptive Data Rate (ADR) mechanism that is responsible for configuring transmission parameters such as Spreading Factor (SF), Bandwidth (BW), and Transmission Power (TP) usually use static, rule-based logic, which does not react well to environmental variability, node mobility, or non-stationary channel conditions [10].

To address these limitations, researchers have used Machine Learning (ML) techniques to optimize LoRaWAN performance in areas such as power control, transmission rate, SF selection, and network scalability [11]. ML’s data-driven nature allows it to capture non-linear dependencies and hidden patterns in wireless environments that rule-based algorithms often overlook. Supervised learning methods like Random Forests (RF) and Support Vector Machines (SVM) have also been found effective [12]. Beyond supervised ML, researchers have also begun applying Reinforcement Learning (RL) for continuous adaptation in unpredictable environments. RL models enabled LoRaWAN devices to autonomously adjust communication strategies through trial-and-error learning [13].

However, many studies remain environment-specific or target specific use cases, which limits the generalizability of the results. González-Palacio et al. [1] highlighted the limitations of traditional models relying solely on distance and frequency, demonstrating the benefits of incorporating environmental variables, such as temperature and humidity, achieving an RMSE of 1.84 dB. Similarly, Anzum et al. [4] improved path loss predictions in LoS and Non-LoS scenarios, achieving an RMSE of 2.74 dB in palm oil plantations. El Chall et al. [14] evaluated path loss in urban and rural Lebanon, showing a reliable coverage of up to 45 km in rural settings. However, many studies remain environment-specific, which limits the generalizability of the results. Callebaut and Perre [15] analyzed urban, forest, and coastal terrains, emphasizing signal diffraction challenges. Bianco et al. [16] demonstrated the viability of LoRa for search and rescue operations but noted significant range reductions in harsh conditions. Other studies, such as those by Jawad et al. [17] and Yang et al. [18], applied machine learning and optimization techniques to enhance accuracy but focused solely on specific use cases. This study highlights the need for comprehensive models incorporating environmental factors while ensuring adaptability across varying conditions and network configurations.

This study highlights the need for comprehensive models incorporating environmental factors while ensuring adaptability across varying conditions and network configurations. This study also investigates the potential of boosting algorithms to improve path loss prediction accuracy in LoRaWANs by incorporating these environmental influences.

The objectives of this study include the following:

1. Address the identified gaps by employing five boosting ML algorithms—Adaptive Boosting (AdaBoost), Extreme Gradient Boosting (XGBoost), Light Gradient Boosting Machine (LGBM), GentleBoost (GB), and LogitBoost (LB)—for accurate path loss prediction in LoRaWAN environments.
2. Evaluate the effect of critical environmental parameters (e.g., temperature, relative humidity, barometric pressure, and PM2.5) on signal attenuation using permutation feature importance.
3. Benchmark the prediction performance of ML models against classical propagation models such as Log-Distance and Okumura-Hata.
4. Conduct a performance vs. complexity analysis by measuring training time, inference time, energy consumption, and model size to assess real-time deployment feasibility.
5. Apply Bayesian Optimization to fine-tune hyperparameters (e.g., learning rate and number of learning cycles) to improve model generalization and predictive accuracy.

This study used an on-premises and physical dataset incorporating various environmental variables, such as temperature, humidity, barometric pressure, and particulate matter. The performance metrics considered in this research were throughput, reduction in latency, jitter, and delay.

The rest of the paper is organized as follows: Section 2 presents an overview of LoRaWAN Technology; Section 3 provides a brief review of related work; Section 4 details the adopted methodology, including data collection and description, PL modeling and transmission parameters, data preparation and preprocessing, boosting algorithms and hyperparameter optimization, cross-validation and evaluation metrics, feature importance assessment, and network simulation setup; Section 5 presents the results and analysis; Section 6 discusses the findings; and Section 7 concludes the paper with future work.

## 2. LoRaWAN Architecture

The networking protocol LoRaWAN has been built upon LoRa’s physical layer, is specifically designed for scalable, low-power, and long-range communication in IoT systems [12]. It features a star-of-stars topology that centralizes traffic from numerous battery-operated End Devices (EDs) through intermediary gateways to a core network server [2]. This structure is optimized for asynchronous, uplink-heavy communication, which suits the nature of most IoT traffic [19], where nodes report data periodically and frequently [3].

The end devices in LoRaWANs communicate directly with nearby gateways using the LoRa modulation. These gateways, in turn, forward the packets without interpreting them to the network server using high-speed backhaul connections like Ethernet or cellular network [12]. The network server handles crucial responsibilities such as data de-duplication, message scheduling, and ADR control. A unique feature of LoRaWANs is the classification of end devices into three operational device classes (A, B, and C), each class is designed for different energy and latency requirements [20]:

- Class A devices, the most energy-conservative, initiate uplinks and then briefly open two receive windows.
- Class B devices synchronize with periodic beacons from gateways, allowing scheduled downlink slots.
- Class C devices are always listening unless transmitting. While highly responsive, they consume significantly more power, making them suitable only for latency-sensitive tasks.

Table 1 presents a quick comparison of the different classes of end nodes in the LoRaWAN architecture.

LoRaWAN communication is generally uplink-dominant due to power-saving strategies, and downlinks are used sparingly to avoid congestion. This is especially because all downlink slots must compete with acknowledgments, ADR commands, and multicast messages [23]. Therefore, scalability is a critical design goal for the LoRaWAN, but it is limited by its reliance on the ALOHA protocol for media access. In dense networks, this contention-based scheme leads to collisions and degraded Packet Delivery Ratios (PDR), particularly in Class A deployments [24].

Furthermore, network performance depends on effective management of radio parameters such as SF, TP, BW, and Coding Rate (CR). These factors are mutually dependent; for example, a higher SF increases communication range but also leads to longer airtime and lower data throughput [12]. However, a major research challenge with the LoRaWAN is packet collision in densely populated networks. Collisions can result in data loss when there are hundreds or thousands of network nodes [25].

### LoRaWAN Datasets

The LoRa research community has observed a lack of large-scale and well-documented datasets that can be used to study resource allocation, interference, collision issues, and the behavior of the LoRaWAN under various conditions [26]. Ref. [27] presented a large-scale LoRaWAN dataset comprising over 86 million transmissions collected from 390 sensors deployed across five floors in a multi-level building over two years. The dataset was subsampled to approximately 1 million balanced transmissions across 1560 sensor-gateway pairs, resulting in 909,414 samples. This dataset has supported both explanatory and predictive modeling of signal quality metrics (ESP, RSSI, and SNR), and represents one of the first real-world, long-term indoor LoRaWAN deployments beyond simulations or small-scale studies. Another study [28] included three LPWAN datasets across rural and urban areas in Belgium. It comprises over 25,000 Sigfox messages from a rural commute area and 14,000 urban Sigfox messages from postal vehicles in Antwerp. Additionally, a LoRaWAN dataset was collected in the same urban area using OCTA-Connect modules. Each message includes GPS coordinates, RSSI, and base station data, enabling spatial correlation for signal strength analysis. Despite some GPS drift in fast-moving conditions, the dataset provided a valuable benchmark for LPWAN localization and performance evaluation in real-world settings.

However, most available datasets are small and often tailored to specific networks or applications as large-scale LoRaWAN deployments are rare. This limits their ability to conduct comprehensive studies across diverse scenarios. Furthermore, using the data gathered by the LoRaWAN for this study is challenging because it is not documented. Despite these drawbacks, the available datasets can be utilized to investigate a range of LoRaWAN network-related concerns [2,29]. For example, these datasets can be used to determine the traffic patterns of LoRaWAN end nodes, the effect of their resource allocation on the network, and the performance of the LoRaWAN itself.

## 3. Related Work

González-Palacio et al. [1] discussed that previous research focused on developing path loss models primarily based on distance and frequency. However, path loss is also affected by variations in weather conditions. Thus, making predictions based solely on conventional and standard methodologies may not account for the significant environmental effects. Consequently, González-Palacio et al. [30] produced a collection of LoRaWAN measurements in Medellín, Colombia. They recorded the path loss, distance, frequency, temperature, relative humidity, barometric pressure, particulate matter, and energy. Therefore, this dataset is highly useful for those aiming to design high-accuracy path loss prediction models. The dataset was implemented using model fittings, including Log-Distance and Multiple Linear Regression (MLR) models that accounted for environmental effects. This analysis demonstrated that incorporating these environmental variables enhanced path loss predictions, yielding an RMSE of 1.84 dB and an R^2^ of 0.917.

Anzum et al. [4] developed an accurate path loss prediction model for LoRaWAN communication at 433 MHz in foliage-rich environments, particularly within Malaysian palm oil plantations. Unlike traditional models that often fail to capture environmental obstructions, such as vegetation, this study introduces a multiwall empirical model adapted to Line-of-Sight (LoS), Non-Line-of-Sight (NLoS), and Obstructed Line-of-Sight (OLoS) scenarios. This study employed a deterministic modeling approach, utilizing empirical data fitting. The authors have collected real-world data from three palm oil plantations in Malaysia under three different propagation scenarios such as LoS, OLoS (one obstruction), and NLoS (two or more obstructions). They used 433 MHz LoRaWAN nodes, deployed at heights of 1.5 m and 3 m, and corresponding gateways, with signal measurements taken at multiple distances. Key parameters such as PL, RSSI, number and type of obstacles (foliage layers), transmission distance, and antenna height were evaluated. The authors enhanced classical models by incorporating a Vegetation Attenuation Factor (VAF) to account for foliage-induced signal degradation. The model demonstrated strong performance, with RMSE values of 2.23 dB, 2.51 dB, and 2.74 dB noted across LoS, OLoS, and NLoS scenarios, respectively.

El Chall et al. [14] developed path loss models for LoRaWAN communications and evaluated them using popular empirical models. Real measurements were taken in both indoor and outdoor settings at urban and rural sites in Lebanon to assess the coverage and performance of the LoRaWAN implementation. The findings demonstrate the accuracy and ease of use of the suggested path loss models in Lebanon and other comparable settings. Urban and rural areas returned coverage ranges of up to 8 km and 45 km, respectively.

Callebaut and Perre [15] utilized urban, forest, and coastal environments to assess coverage and path loss. Diffraction effects cause greater signal loss than a traditional star-of-stars topology because the terminals are usually positioned at low altitudes. They also considered censored data to estimate realistic path loss parameters. Because 80% of the transmitted packets were successfully received at approximately 200 m, the packet error ratio was calculated using the expected path loss characteristics to assess the performance of the point-to-point networks. In addition, a range of more than 4 km was observed in the LoS situation. Even with heavily forested terrain and complex urban radio propagation, a maximum range of 1 km has been attained.

Bianco et al. [16] presented and examined a LoRa-based system for search and rescue operations. Measurements were used to derive radio path loss models for body-worn LoRa devices in harsh mountain environments. Due to its extended radio range, researchers have found that Low-Power Wide-Area Network (LPWAN) technology is highly promising for search and rescue applications. Even though the range of LoRa drops from kilometers to hundreds of meters in the testing environment. The researchers determined that at least 50% of the transmitted packets can be received at distances roughly five times greater than with gold standard technologies, such as ARVA.

Masek et al. [26] presented measurements of various operational aspects of LoRaWAN communication technology collected in Brno, Czech Republic. This dataset aims to assist researchers who lack access to LoRaWAN devices. It provided data collected from 303 outdoor test locations. These locations are transmitted to up to 20 gateways operating in the 868 MHz band. The data encompassed a range of urban landscapes, enabling comparison and analysis across diverse environments. Furthermore, they developed a prototype with a Microchip RN2483 LPWAN LoRaWAN technology transceiver module for field measurements. Researchers have demonstrated the relationship between the Received Signal Strength Indicator (RSSI) and the Signal-to-Noise Ratio (SNR) and the distance from the closest gateway.

Using the MATLAB curve-fitting tool, Jawad et al. [17] proposed two novel path loss models for a ZigBee WSN in a farm field. Polynomial and exponential functions were the foundation for developing the two path loss models. Both functions were merged with Particle Swarm Optimization (PSO) to determine the ideal coefficients of the functions that would produce the POLY-PSO algorithms. The obtained values surpassed those of earlier state-of-the-art PL models. The findings demonstrate that the hybrid EXP-PSO and POLY-PSO models significantly increased the regression line’s coefficient of determination, with the Mean Absolute Error (MAE) for EXP-PSO and POLY-PSO being 1.6 and 2.7 dBm, respectively.

Yang et al. [18] proposed a feature selection strategy to improve the accuracy and generalization of ML approaches for predicting path loss and delay spread in air-to-ground millimeter-wave channels. The researchers employed transfer learning techniques to predict path loss with little data. They compared the Okumura–Hata and COST-231 Hata models with the suggested methods for path loss prediction. The lognormal distribution is used as a comparison or baseline model to predict the delay spread. The novel techniques, therefore, had lower RMSE values than contrast models based on data produced by the ray-tracing software.

Aernouts et al. [28] presented a well-documented dataset covering urban and rural areas in Belgium. The study captured LoRaWAN transmissions using GPS-tagged mobile devices over Proximus’s national LoRaWAN network. Data included over 14,000 transmissions in urban Antwerp and 25,000 in a rural corridor between Antwerp and Ghent, recorded between November 2017 and February 2018. The authors have analyzed signal strength using parameters such as RSSI across various terrains using Proximus’s nationwide LoRaWAN network. Devices sent GPS-tagged messages at regular intervals, allowing for precise correlation between signal quality and location. The basic KNN fingerprint algorithm has been used to determine the optimal K value for each set. The rural Sigfox dataset achieved the lowest mean location error of 214.58 m, and the urban Sigfox dataset had a mean error of 688.97 m. For the urban LoRaWAN dataset, the mean error was 398.4 m. These results demonstrated a significant impact of environmental complexity on localization accuracy.

A summary of related work is presented in Table 2.

As presented in Table 2, the measurement and path loss modeling studies for the LoRaWAN mainly focused on distance, frequency, and antenna height. However, previous studies have shown that environmental factors such as temperature, relative humidity, barometric pressure, and particulate matter influence path loss variability. These environmental effects have not been measured in the available LoRaWAN datasets. Therefore, this research adopts a comprehensive LoRaWAN measurement study conducted over four months in an urban environment in Medellín, Colombia, by González-Palacio et al. [1].

## 4. Methodology

### 4.1. Data Collection and Description

This study used an open-access dataset provided by González-Palacio et al. [5]. The dataset includes critical environmental parameters such as temperature, relative humidity (rh), barometric pressure (bp), and particulate matter (PM2.5) concentrations (µg/m^3^). Transmission parameters, including transmitter power ($P_{tx}$), antenna gains ($G_{tx}$ and $G_{rx}$), transmission and receiver losses ($L_{tx}$ and $L_{rx}$), and received signal strength indicator (rssi), were also recorded.

### 4.2. PL Modeling and Transmission Parameters

This study used PL as the primary measure to quantify signal attenuation in the LoRaWAN. PL is a critical parameter in wireless communication as it reflects the reduction in signal strength from the transmitter to the receiver. Path loss is calculated by considering the following equation (Equation (1)):

(1) $$PL=P_{tx}+G_{tx}-L_{tx}+G_{rx}+L_{rx}-RSSI$$

where

- $P_{tx}$ represents the initial strength of the signal emitted by the end node in dBm. It is a critical parameter that defines the baseline signal power before any losses or gains occur in the transmission process.
- $L_{tx}$ account for the signal attenuation caused by the cables and connectors used in the transmission systems.
- $G_{tx}$ reflects the antenna’s ability to amplify the signal before it is transmitted into the environment.
- $L_{rx}$ represent the attenuation of the signal in the cables and connectors on the gateway side.
- $G_{rx}$ captures the additional amplification provided by the gateway’s antenna.

In Equation (1), we added $L_{tx}$ by considering transmission system losses, such as those incurred by cables and connectors. These losses are critical factors in practical wireless communication scenarios, as highlighted by Mahmood et al. [31]. Therefore, $L_{tx}$ ensures the model’s accuracy, which is essential for the proper measurement of transmission power, reliable link budget analysis, and energy efficiency in LoRaWAN.

### 4.3. Data Preparation and Preprocessing

A comprehensive preprocessing process has been followed to prepare a dataset for implementation. First, the dataset was randomized to eliminate any sequential biases that could affect the learning process. The randomized dataset was split into training and testing subsets, with 80% of the data allocated for training and 20% for testing. This split ensured that the model had sufficient data for training while retaining a substantial portion for unbiased evaluation.

Normalization was applied to all continuous input variables, including temperature, relative humidity, barometric pressure, and RSSI, using z-score normalization. This step was essential to mitigate the disproportionate influence of variables with differing magnitudes or measurement units on model training. The normalization followed the standard formula (Equation (2)):

(2) $$X_{\mathrm{normalized}}=\frac{X-\mu }{\sigma }$$

where *X* refers to the original, unnormalized value of the variable (feature) for a given observation in the dataset, μ is the mean, and *σ* is the standard deviation of each feature.

The dataset from González-Palacio et al. [5] did not categorize path loss values into classes. Therefore, PL values were classified into two categories based on a computed threshold (M), representing the mean PL across all observations. This binary classification simplified the prediction task and enabled the application of boosting algorithms designed for supervised learning tasks. Equation (3) shows the threshold calculation.

(3) $$M=\frac{\sum_{i=1}^{n}PL_{i}}{n}$$

where *n* is the total number of PL observations. Observations with PL≤M were assigned to Class 0 (low path class), and those with PL>M were labeled as Class 1 (high path loss).

Missing values were addressed using median imputation following Equation (4) to ensure incomplete records did not compromise the models’ performance.

(4) $$X_{filled}=\left\{\begin{array}{c} x_{i},\;\;x\;is\;not\;missing\\ \tilde{X},\;\;x\;is\;missing \end{array}\right.$$

where $\tilde{X}$ denotes the median of the feature vector. These preprocessing steps resulted in a clean, balanced dataset that was then used for further evaluation.

### 4.4. Boosting Algorithms and Hyperparameter Optimization

The five boosting algorithms employed in this study were chosen for their proven ability to handle nonlinear relationships and high-dimensional feature spaces, which are common in wireless communication environments influenced by multiple environmental and physical factors. All boosting algorithms were implemented using MATLAB’s (R2024b) ensemble learning framework. AdaBoost, GentleBoost, and LogitBoost were trained using the fitcensemble function with their respective methods “AdaBoostM1”, “GentleBoost”, and “LogitBoost” to make them well-suited for binary classification tasks based on path loss thresholds. These models adaptively adjust sample weights to emphasize misclassified instances, with GentleBoost applying smoother weight updates for enhanced stability and LogitBoost optimizing the binomial log-likelihood for improved probabilistic classification. In contrast, XGBoost and LightGBM were implemented using fitrensemble with the “LSBoost” method, utilizing regression-based boosting to capture complex nonlinear relationships in path loss behavior. XGBoost incorporated depth control with a templateTree structure, improving its ability to generalize across different environmental conditions. Each algorithm was fine-tuned using Bayesian Optimization to enhance model accuracy and generalization. The optimization process minimized the RMSE of the predictions on a validation set following the general form given in Equation (5).

(5) $$\underset{\theta \in H}{\min}\;\mathrm{RMSE}=\sqrt{\frac{1}{n}\overset{n}{\underset{i=1}{\sum }}{\left(y_{i}-{\hat{y}}_{i}(\theta )\right)}^{2}}$$

where *θ* is the set of hyperparameters, $y_{i}$ is the true label, and ${\hat{y}}_{i}(\theta )$ is the model prediction using parameters *θ*. Hyperparameters such as the number of boosting iterations (set between 10 and 200) and the learning rate (ranging from 0.01 to 1.0) were optimized. In the case of XGBoost, an additional parameter, maximum tree depth, was included to control the model’s complexity. Each model was evaluated using 5-fold cross-validation, and the optimization was conducted using a maximum of 30 objective function evaluations for consistency and computational efficiency, as recommended by MathWorks.

### 4.5. Cross-Validation and Evaluation Metrics

To ensure robust evaluation of the proposed boosting models, this study employed a 5-fold cross-validation technique. The dataset was randomly partitioned into five equal parts, where, in each iteration, one fold was used for testing while the remaining four were used for training. This process was repeated five times to ensure that each subset served as the test set exactly once. This stratified validation method effectively reduced overfitting, improved the models’ generalizability, and provided a more reliable performance estimate.

Model performance was evaluated across multiple dimensions, including network-level indicators such as throughput, latency, jitter, and delay per node. Additionally, critical metrics such as prediction accuracy and the number of nodes remaining alive after simulation were used to measure the effectiveness of the boosting algorithms in real-world LoRaWAN deployments. Quantitatively, model performance was assessed using the standard regression metrics defined in Equations (6)–(9). The Root Mean Squared Error (RMSE), Mean Squared Error (MSE), Mean Absolute Error (MAE), and Coefficient of Determination (R^2^) were used to quantify the accuracy and variability of the predicted path loss values. These metrics are well-established in the statistical learning and signal processing literature and are commonly applied to evaluate regression-based prediction models [1,30].

(6) $$\mathrm{RMSE}=\sqrt{\frac{1}{N}\overset{N}{\underset{i=1}{\sum }}{\left(PL_{\mathrm{measured},i}-PL_{\mathrm{predicted},i}\right)}^{2}}$$

(7) $$\mathrm{MSE}=\frac{1}{N}\overset{N}{\underset{i=1}{\sum }}{\left(PL_{\mathrm{measured},i}-PL_{\mathrm{predicted},i}\right)}^{2}$$

(8) $$\mathrm{MAE}=\frac{1}{N}\overset{N}{\underset{i=1}{\sum }}|PL_{\mathrm{measured},i}-PL_{\mathrm{predicted},i}|$$

(9) $$R^{2}=1-\frac{\sum_{i=1}^{N}{\left({PL}_{measured,\;i}-{PL}_{predicted,\;\;i}\right)}^{2}}{\sum_{i=1}^{N}{\left({PL}_{measured,\;i}-\bar{{PL}_{measured}}\right)}^{2}}$$

The performance metrics defined in Equations (6)–(9) were applied across all five boosting algorithms to provide a multidimensional evaluation framework that balances sensitivity to error magnitude, variance, and absolute deviation. In addition to the above, the performance of each boosting model in terms of RMSE and R^2^ score was compared against the classical Log-Distance and Okumura-Hata theoretical PL models. This comparison was performed under urban propagation conditions, aligning with the real-world environmental context from which the dataset was collected. For the Log-Distance path loss model, the predicted PL at distance d was computed using the following:

(10) $$PL\left(d\right)=PL\left(d_{0}\right)+10\cdot n\cdot \log_{10}\left(\frac{d}{d_{0}}\right)$$

where

- $PL(d_{0})$ is the path loss at the reference distance (set as 40 dB).
- n is the path loss exponent (3.5 for urban areas).
- d is the distance between the transmitter and receiver (in meters).
- $d_{0}$ is the reference distance (1000 m).

For the Okumura-Hata model, which is widely accepted for urban scenarios, the following empirical formula (Equation (11)) was applied:

(11) $$L=69.55+26.16\cdot \log_{10}(f)-13.82\cdot \log_{10}(h_{t})-c(h_{r})+\left[44.9-6.55\cdot \log_{10}(h_{t})\right]\cdot \log_{10}(d)$$

where

- L is the path loss in dB.
- f is the frequency in MHz (905 MHz in this study as given in the dataset).
- $h_{t}$ and $h_{r}$ are the heights of the transmitter and receiver, respectively (40 m and 5 m as given in the dataset).
- d is the distance between the transmitter and receiver (in meters).
- $c(h_{r})$ is the receiver height correction factor for urban areas, given by the following:
  (12) $$c(h_{r})=3.2\cdot {\left[\log_{10}(11.75\cdot h_{r})\right]}^{2}-4.97$$

### 4.6. Feature Importance Assessment

Permutation importance was utilized to evaluate the contribution of each environmental feature to the accuracy of PL prediction. This model-agnostic method, originally proposed by Engmann [32], quantifies feature relevance by measuring the decrease in prediction performance when a specific feature’s values are randomly permuted. This study applied the permutation importance approach by first computing the baseline model accuracy using unmodified input features. Then, for each feature, its values were independently shuffled across all samples, and the model’s predictive accuracy was recalculated. The importance score was defined as the drop in accuracy caused by this permutation. For each feature *i*, importance is computed as follows:

(13) $${\mathrm{Importance}}_{i}=A_{\mathrm{base}}-A_{i}$$

where

- $A_{\mathrm{base}}$ is model accuracy on the original dataset.
- $A_{i}$ is model accuracy on the dataset where feature *i* has been randomly shuffled.

Accuracy itself was derived from the confusion matrix as follows:

(14) $$Accuracy=\frac{\sum diag\left(C\right)}{\sum C}\times 100$$

where C is the confusion matrix of true vs. predicted labels. Since the original models were trained for regression, the predictions and true labels were rounded before computing classification-based accuracy. This strategy enables an interpretable and quantifiable understanding of which environmental parameters, such as barometric pressure, humidity, temperature, and PM2.5, influence signal attenuation in LoRaWAN.

### 4.7. Network Simulation Setup

A MATLAB-based simulation environment was developed to emulate the behavior of a LoRaWAN under realistic conditions. The simulated network comprised 500 nodes deployed within a 2500 m × 2500 m area. The chosen area size represents a typical deployment environment for the urban scenario, as represented in the dataset. An initial energy value (10 Joules) for WSN nodes is derived from commercial devices like LoRaWAN-enabled sensors, which generally have energy budgets in the 5–20 Joules range as used by Elshrkawey et al. [33] and Engmann et al. [32]. The simulation was run for a total of 600 s, which allowed sufficient time for evaluating dynamic network behavior and capturing time-sensitive performance metrics such as delay, jitter, and energy consumption. The spatial distribution of nodes within the simulation area was randomized to ensure that the test environment represented a wide range of deployment scenarios.

The process begins with sampling input data, such as SNR, distance, and packet size, and initializing metrics for throughput, latency, jitter, and delay. Nodes are randomly assigned transmission intervals to simulate realistic asynchronous communication. Based on an ML model, energy efficiency predictions categorize nodes into high or low efficiency, impacting their performance metrics. High-efficiency nodes are expected to show increased throughput and reduced latency, jitter, and delay compared to low-efficiency ones. The simulation calculates energy consumption per transmission and reception, factoring in the node’s distance to the gateway. Over the simulation period, energy consumption depletes node energy levels. Nodes cease communication once energy is exhausted, reflecting real-world resource constraints. The simulation outputs key performance metrics and logs the number of alive nodes and their remaining energy levels. Adhering to regulatory constraints, such as the duty cycle limit of 0.662%, was given special attention. Transmission intervals and frame sizes were adjusted to ensure compliance, resulting in a realistic simulation of LoRaWAN operations. The parameters of the test network are shown in Table 3.

These test network parameters were chosen to balance the computational efficiency and complexity needed to simulate a moderately dense WSN environment

## 5. Results and Analysis

This study conducted comprehensive evaluations to assess the performance and practical implications of boosting algorithms. The analysis encompassed five key dimensions: (1) network performance evaluation, including throughput, latency, jitter, and delay; (2) performance vs. complexity trade-off by comparing each model in terms of training time, inference time, and model size; (3) energy efficiency assessment, measured by the number of alive nodes remaining after network simulation; (4) feature impact analysis using permutation importance to determine which environmental parameter (e.g., temperature, humidity, barometric pressure, PM2.5) most significantly affected path loss; and (5) predictive accuracy assessment, using model accuracy, RMSE, MAE, MSE, and R^2^. These evaluations provided a multi-dimensional understanding of each algorithm’s strengths, weaknesses, and suitability for real-world deployment in LoRaWAN environments.

### 5.1. Network Performance Analysis

The network performance of each boosting model was evaluated across four key metrics: throughput, latency, jitter, and end-to-end delay, measured per node. Table 4 presents a comparative analysis of the average performance demonstrated by each model across all evaluated metrics. The results were collected from a simulation evaluating each model under identical conditions with 500 nodes.

LightGBM demonstrated strong efficiency in handling data. It recorded the highest average throughput among all models. GentleBoost showed slightly lower throughput but still outperformed AdaBoost, LogitBoost, and XGBoost. AdaBoost achieved the lowest average latency. It responded faster than the other models in message delivery. GentleBoost had almost the same latency. Jitter was lowest in AdaBoost and GentleBoost. These models maintained more consistent transmission intervals. XGBoost had the highest jitter. It showed more variation in packet delivery times. Delay values were similar across all models. AdaBoost recorded the lowest delay, showing the best performance in minimizing end-to-end transmission time.

### 5.2. Performance vs. Complexity Analysis

A comprehensive performance versus complexity analysis was conducted to evaluate the practical deployment viability of the boosting models in LoRaWAN scenarios. This analysis considers multiple dimensions of computational cost, including training time, inference time, energy consumption, and model size, in addition to model accuracy. Table 5 summarizes these metrics across all five models. The AdaBoost model exhibited the highest training time, at 492.60 s, and the highest inference time, at 2.75 milliseconds. Additionally, it required 202.68 MB of memory. XGBoost, on the other hand, offered the highest accuracy of 89.10%. It demonstrated better efficiency than AdaBoost, with a training time of 297.02 s and an inference time of 1.03 milliseconds. However, its model size reached 250.09 MB.

LightGBM achieved a near-peak accuracy of 89.17% and outperformed others in computational efficiency. It recorded the lowest training time, 190.36 s, and the shortest inference time, 0.90 milliseconds. The model size, however, was the largest, at 291.89 MB, which may pose storage concerns on memory-constrained devices. The GentleBoost model achieved an accuracy of 88.85%, with moderate training times (316.14 s) and inference times (2.31 milliseconds). The model size was 222.89 MB. These results suggest a balanced trade-off between performance and complexity. LogitBoost produced 88.69% accuracy with a training time of 281.94 s and an inference time of 1.51 milliseconds. It had the smallest model size, 176.29 MB.

### 5.3. Energy Efficiency Analysis

**Figure 1:**
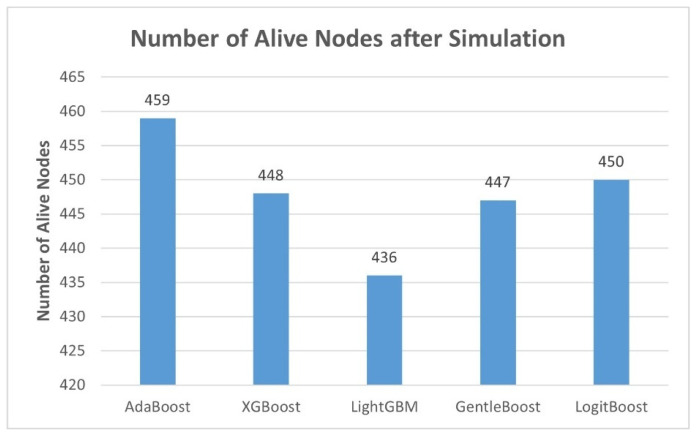
Energy efficiency analysis of boosting machine learning models in LoRaWAN.

Figure 1 illustrates the energy efficiency analysis across the evaluated boosting models by showing the number of alive nodes remaining after the simulation. In energy-constrained LoRaWAN environments, a higher number of alive nodes directly reflects lower energy depletion during communication processes and better overall energy optimization of the model. AdaBoost demonstrates the highest energy efficiency among all models, retaining 459 alive nodes. LogitBoost and XGBoost follow closely, with 450 and 448 alive nodes, respectively, while GentleBoost maintains 447. LightGBM results in the fewest alive nodes at 436.

### 5.4. Feature Impact Analysis Using Permutation Importance

**Figure 2:**
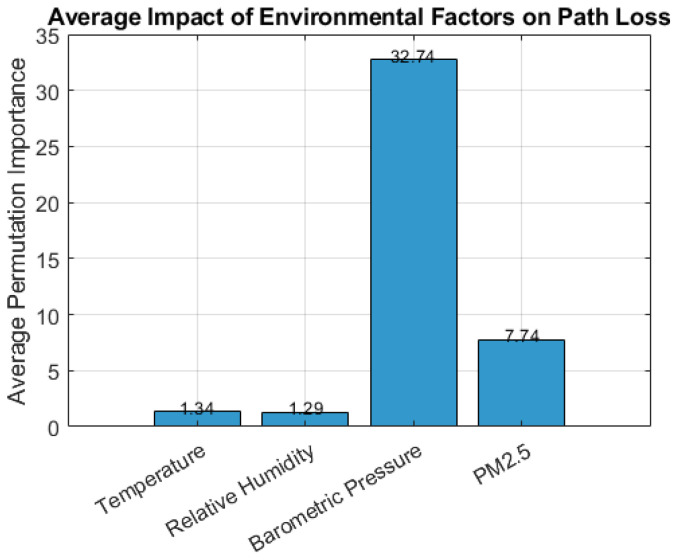
Feature impact analysis using permutation importance.

A permutation importance approach was utilized to evaluate the relative contribution of environmental features in influencing path loss predictions across all boosting models. This method quantifies the importance of each feature by measuring the decrease in model performance when that feature’s values are randomly shuffled, thereby breaking the relationship between the feature and the target variable. Figure 2 presents the average permutation importance scores aggregated across all five boosting models. Among the features analyzed, barometric pressure exhibited the highest influence on path loss, with an average impact score of 32.74. This indicates that variations in barometric pressure contribute most substantially to fluctuations in signal attenuation.

PM2.5 concentration followed with a moderate average importance of 7.74, implying a secondary but notable influence on signal degradation. In contrast, temperature and relative humidity showed lower average impact values of 1.34 and 1.29, respectively, suggesting a relatively minimal effect on path loss in the examined urban LoRaWAN scenario. These findings reinforce that barometric conditions and particulate matter levels are critical environmental factors of LoRaWAN.

### 5.5. PL Prediction Accuracy Assessment of Boosting ML Models

**Figure 3:**
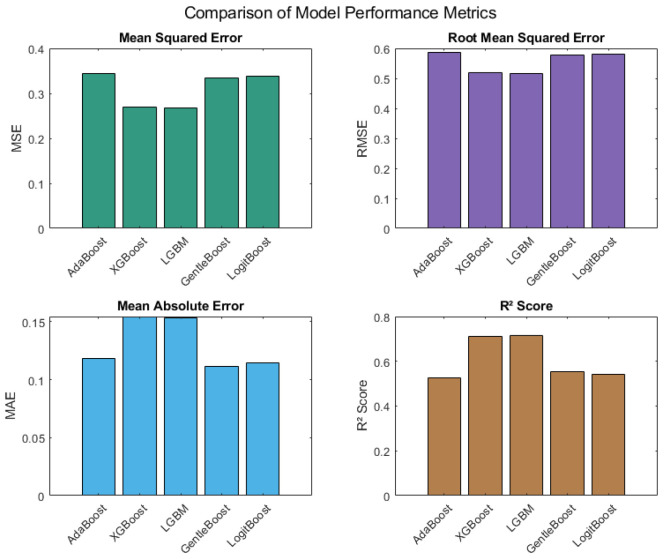
Predictive accuracy assessment of boosting ML models.

Figure 3 compares the five boosting models based on four key predictive performance metrics: MSE, RMSE, MAE, and R^2^. The LightGBM model attained the lowest MSE value of 0.2669 and the lowest RMSE of 0.5166, indicating superior error minimization. It also achieved the highest R^2^ score of 0.7151, suggesting the strongest ability to explain variance in the predicted path loss. XGBoost followed with an MSE of 0.2694, RMSE of 0.5190, and R^2^ of 0.7097. GentleBoost and LogitBoost returned RMSEs of 0.5778 and 0.5820, respectively, and R^2^ scores of 0.5541 and 0.5411. AdaBoost showed relatively higher MSE and RMSE values (0.3442 and 0.5867, respectively) and a lower R^2^ of 0.5261 compared to LightGBM and XGBoost. However, AdaBoost achieved the lowest MAE value (0.1185), reflecting good average error magnitude. These findings, illustrated in Figure 3, offer a comprehensive quantitative basis for comparing each model’s predictive performance under the tested conditions.

### 5.6. PL Prediction Accuracy: Boosting vs. Other ML Models

RMSE and R^2^ score metrics were used to compare the predictive accuracy of the five boosting algorithms employed in this study with those of ML models reported in existing studies. Table 6 presents a side-by-side comparison that includes Multiple Linear Regression (MLR) from González-Palacio et al. [1], as well as Artificial Neural Networks (ANN), Random Forests (RF), and Support Vector Regression (SVR) from González-Palacio et al. [30]. Table 5 shows that the RF model achieved the lowest RMSE of 1.566 dB and the highest R^2^ of 0.9389 among the referenced models. Similarly, SVR and ANN also recorded competitive RMSE values of 1.626 dB and 1.613 dB, respectively, with corresponding R^2^ scores of 0.9342 and 0.935.

In this study, boosting-based models demonstrated notably improved performance in terms of prediction accuracy. Specifically, LightGBM and XGBoost achieved the lowest RMSE values of 0.5166 dB and 0.5190 dB, respectively, surpassing all prior models in error minimization. These were followed by LogitBoost (0.5820 dB), GentleBoost (0.5778 dB), and AdaBoost (0.5867 dB). Regarding the R^2^ score, XGBoost and LightGBM produced the highest values among the boosting models, with values of 0.7097 and 0.7151, respectively. These findings indicate a meaningful reduction in prediction error. However, this observed improvement in RMSE among the boosting models is accompanied by a notable decrease in R^2^ compared to traditional models such as RF and SVR. This divergence reflects a critical trade-off between minimizing point-wise error and explaining overall variance in predictive modeling. RMSE, being a scale-dependent metric, captures the average magnitude of residuals but does not assess how well the model accounts for variability in the target variable.

Conversely, R^2^ measures the proportion of variance in the observed data explained by the model, making it sensitive to both dispersion and model structure. Boosting algorithms, such as AdaBoost, LightGBM, and XGBoost, are specifically designed to iteratively reduce errors by optimizing loss functions, including squared error. As a result, these models often achieve superior RMSE performance. However, they may not yield high R^2^ scores if the variance structure of the dataset is not effectively captured. This behavior is well-documented in the ensemble learning literature, where boosting emphasizes bias reduction at the expense of variance explanation under certain conditions [34,35,36].

The boosting models succeeded in reducing absolute prediction errors. Still, they failed to match the R^2^ levels observed in models like RF, which are known for capturing complex variance through bagging and feature randomness [35]. The discrepancy between RMSE and R^2^ thus highlights an important modeling insight: boosting techniques are optimal when the primary objective is precise prediction. At the same time, models with higher R^2^ are better suited for interpretability and variance attribution [37].

### 5.7. PL Prediction Accuracy: Boosting vs. Theoretical Models

The Log-Distance and Okumura-Hata theoretical models are compared with boosting ML models in predicting PL. These models are widely adopted in wireless communication studies for estimating signal attenuation based on distance and environmental parameters. The Log-Distance model is commonly used in empirical radio propagation scenarios, offering flexibility across different terrain types. In contrast, the Okumura-Hata model is a well-established empirical model specifically designed for urban environments, taking into account factors such as frequency, base station height, and distance. Table 7 presents the RMSE and R^2^ Scores for each of the five boosting algorithms evaluated in this study, compared to the Log-Distance and Okumura-Hata theoretical models.

The RMSE values for the boosting models are significantly lower than those of the theoretical models. The theoretical models exhibited relatively high R^2^ scores, which can be attributed to their inherent structure, which closely aligns with the large-scale path loss trends in idealized conditions. Models like Log-Distance and Okumura-Hata are structured to fit large-scale signal trends using log-distance formulations [38], which closely match the global variance patterns present in the dataset. As a result, they explain a larger portion of the variance, leading to a higher R^2^. However, these models overlook local signal fluctuations caused by environmental factors such as humidity, temperature, and particle density. This leads to higher absolute errors, reflected in their poor RMSE. Boosting models capture these fine-grained variations through feature learning [34,36], which significantly reduces RMSE but may not necessarily maximize R^2^, as the learned variance is more distributed and less aligned with the dominant global trend.

These results highlight the improved predictive precision of boosting ML approaches in modeling complex environmental conditions that affect signal propagation in LoRaWAN.

## 6. Discussion

This study extensively evaluated boosting ML models to assess their effectiveness in predicting path loss in LoRaWAN environments under realistic environmental conditions. The boosting ML models were thoroughly benchmarked using RMSE, R^2^, MAE, and MSE alongside network performance and computational complexity indicators. LightGBM achieved the lowest RMSE (0.5166) and highest R^2^ (0.7151). Furthermore, LightGBM not only demonstrated the highest throughput and lowest inference time but also required the least training time among the ensembles. This makes it particularly attractive for real-time and resource-constrained deployments. However, this advantage came at the cost of a slight compromise in energy efficiency, as evidenced by the lower number (436) of alive nodes after simulation.

LightGBM consistently outperformed the other boosting models in prediction and computational metrics due to its leaf-wise tree growth strategy, histogram-based decision tree learning, and Exclusive Feature Bundling (EFB). Unlike traditional boosting models, such as AdaBoost or XGBoost, which grow trees level-wise, LightGBM grows trees leaf-wise, selecting the leaf with the maximum split gain to grow [32]. This strategy enables the model to converge more quickly and achieve lower loss with fewer iterations, thereby directly improving PL prediction accuracy, as observed in this study. Furthermore, LightGBM applies a Gradient-Based One-Side Sampling (GOSS) technique, which retains instances with large gradients and randomly samples those with slight gradients. This approach significantly reduces the number of data points used in training while preserving accuracy, thereby reducing training time and inference delay [39,40]. The histogram-based algorithm used in LightGBM further contributes to speed by discretizing continuous feature values into bins. This significantly reduces memory usage and computation time compared to the greedy algorithms used in models like XGBoost [39,41]. Additionally, EFB helps address sparse features by bundling mutually exclusive features into a single feature, thereby significantly reducing dimensionality and enhancing performance [39].

AdaBoost achieved the highest number of alive nodes (459), indicating strong energy-aware performance, and maintained a consistent throughput with the lowest average delay fluctuation. However, it exhibited the longest training time (492.6 s) and the lowest throughput. XGBoost offered a balance. It shows lower training time than AdaBoost, moderate latency, and good performance in network metrics. Hence, it strikes a middle ground between LightGBM speed and AdaBoost’s energy preservation. Performance vs. complexity analysis further highlighted that models with faster inference times (LightGBM and XGBoost) had increased memory consumption and energy trade-offs.

Permutation importance analysis consistently revealed barometric pressure as the most dominant factor influencing PL in LoRaWANs. This observation aligns with physical propagation principles and validates the inclusion of environmental variability in ML-based PL modeling. Unlike theoretical models such as Log-Distance and Okumura-Hata, which rely on static propagation conditions, boosting models effectively learn from dynamically varying sensor inputs. Table 6 confirms that both theoretical models produced RMSEs significantly above 2.5 dB, compared to sub-0.6 dB RMSEs achieved by ML models. When compared to ML models proposed in prior works, such as ANN, RF, and SVR, the boosting models in this study demonstrated superior RMSEs and comparable R^2^ values, despite relying on a leaner set of input features and requiring less computational overhead. This reflects a more practical application potential for network scenarios requiring prediction precision and efficiency.

The principal outcomes derived from this study are summarized as follows:

- LightGBM emerged as the most accurate and computationally efficient model, with the lowest RMSE (0.5166), highest throughput (151.54), and fastest inference (0.90 ms).
- AdaBoost offered exceptional energy efficiency, maintaining the highest number of alive nodes (459), but it incurred higher training costs and longer inference delays.
- Performance vs. complexity analysis confirmed that lower inference times often came with increased memory and energy costs.
- Permutation importance confirmed barometric pressure as the dominant environmental factor affecting path loss, far surpassing humidity, temperature, and PM2.5.
- Boosting models substantially outperformed theoretical models (Log-Distance, Okumura-Hata) and provided better accuracy than the ANN, RF, and SVR models reported in the literature.

## 7. Conclusions

This study comprehensively evaluated the effectiveness of boosting ML models for path loss prediction in the LoRaWAN using real-world environmental data. The analysis incorporated a multidimensional assessment, including predictive accuracy, network performance, computational complexity, energy efficiency, and environmental factor sensitivity, as determined through permutation importance.

LightGBM consistently outperformed other models in predictive performance and computational efficiency among the evaluated models. It achieved the lowest MSE, RMSE, and MAE while recording the highest R^2^ score, demonstrating superior generalization capabilities. Furthermore, LightGBM exhibited the fastest inference time and lowest training time. Comparative analysis with RF, ANN, MLR, SVR, and theoretical models Log-Distance and Okumura-Hata revealed that the boosting-based ML models, particularly LightGBM and XGBoost, significantly enhance prediction accuracy. The permutation importance analysis further identified barometric pressure and PM2.5 as the most influential environmental features affecting signal attenuation.

This study highlights the potential of boosting ML algorithms to provide robust, efficient, and adaptive solutions for path loss estimation in dynamic IoT environments. Future work will broaden the experimental scope by exploring neural network architectures, such as Long Short-Term Memory (LSTM) for time-series path loss prediction to provide a comparative perspective against the boosting models. Furthermore, the models will be validated on multi-regional datasets containing diverse environmental conditions, such as coastal, desert, and rural settings.

## Figures and Tables

**Table 1:** Different classes of end nodes in LoRaWAN architecture.

End Node Class	Description
Class A	Run on battery power and use very little energy. These end nodes are bidirectional and can manage two available receive windows when they receive an acknowledgment from the network server [20].
Class B	They have bidirectional communication and are also battery-operated. Although these end nodes have several receive windows and are integrated with a beacon frame de-livered by the gateway at the end of a particular time, they permit unicast and multicast transmission [21].
Class C	These end nodes in the LoRaWAN are designed to always be available to receive messages. Class C devices keep their receive windows open continuously, except when actively transmitting data. This continuous listening makes them highly responsive but more power-intensive [22].

**Table 2:** Summary of related work.

Reference	Purpose	Parameters Measured	Frequency/Bandwidth	Observations
Anzum et al. [4]	Empirical path loss prediction model for oil palm plantations to evaluate LoRaWAN propagation characteristics in dense foliage	RSSI, path loss exponent, trunk, and canopy attenuation values	433 MHz, BW 125 kHz to 500 kHz	Proposed a multiwall model achieving high prediction accuracy with RMSE of 2.74 dB. Verified against Weissberger and ITU-R models
González-Palacio et al. [1]	LoRaWAN path loss measurements in urban environments by considering environmental effects	Distance, RSSI, SNR, temperature, humidity, barometric pressure, PM2.5, frame length	903.9–905 MHz, BW 125 kHz	Included environmental variables to improve path loss modeling, achieving RMSE of 1.84 dB and R^2^ = 0.917
Masek et al. [26]	LoRaWAN measurements in urban environments by focusing on network characteristics and coverage	RSSI, SNR, distance, temperature, power consumption, message size, SF, long-term fluctuations	868 MHz, BW 125 kHz (Europe)	Data from 311 locations; outage probability 5.15%; packet delivery ratio 83%; SNR shows stronger variability
El Chall et al. [14]	Derived path loss models for the LoRaWAN in indoor, urban, and rural environments using empirical data	Distance, RSSI, SNR, antenna height, wall/floor losses, shadow fading	868 MHz, BW 125 kHz	Proposed accurate PL models with ranges up to 8 km (urban) and 45 km (rural); indoor floor loss decreases logarithmically
Callebaut et al. [15]	Model and evaluate point-to-point LoRa path loss in urban, coastal, and forested environments	RSSI, SNR, distance, spreading factor, packet error ratio, antenna height, censored data handling	868 MHz, BW 125 kHz	The maximum range is 4 km (coastal) and 1 km (urban); the proposed ML-based model outperformed OLS and existing models
Bianco et al. [16]	Evaluated the feasibility of the LoRaWAN for search and rescue operations in harsh mountain environments	RSSI, SNR, distance, path loss in canyon and snowy conditions, body shadowing, and antenna height	868 MHz, BW 125 kHz	LoRa achieved 5× greater range than ARVA in snowy environments, with a PDR > 50% developed localization algorithm for search and rescue operations
Aernouts et al. [28]	Created datasets for fingerprinting localization in large urban and rural areas for the LoRaWAN and Sigfox	RSSI, GPS coordinates, base station IDs, transmission time, spatial spread	Sigfox: 868 MHz, LoRaWAN: 868 MHz	Rural Sigfox mean error: 214.58 m; Urban Sigfox: 688.97 m; Urban LoRaWAN: 398.4 m. Dense urban datasets enable lower errors
González-Palacio et al. [30]	Developed ML-based models to predict combined path loss and shadowing for energy efficiency in the LoRaWAN	Distance, RSSI, SNR, spreading factor, temperature, humidity, barometric pressure, PM2.5	902–928 MHz, BW 125 kHz	ANN model achieved RMSE = 1.57 dB; R^2^ = 0.94; energy improvements up to 43% with an enhanced Adaptive Data Rate (ADR) protocol

**Table 3:** Parameters used in network simulation.

Parameter	Value
Number of nodes	500
Area	2500 m × 2500 m
Initial energy per node	10 J
Energy required for transmission	0.05 J
Energy required for reception	0.02 J
Simulation Time	600 s

**Table 4:** Average performance demonstrated by boosting models.

Model	Throughput (Packets/Node)	Latency (s)	Jitter (s)	Delay (s)
AdaBoost	149.32	0.4380	0.2550	0.4167
GentleBoost	150.75	0.4388	0.2553	0.4168
LightGBM	151.54	0.4399	0.2585	0.4180
LogitBoost	149.51	0.4398	0.2599	0.4178
XGBoost	149.37	0.4450	0.2645	0.4233

**Table 5:** Performance vs. complexity analysis of boosting machine learning models.

Model	Accuracy (%)	Training Time (s)	Inference Time (MS)	Model Size (MB)
AdaBoost	88.20	492.60	2.75	202.68
XGBoost	89.10	297.02	1.03	250.09
LightGBM	89.17	190.36	0.90	291.89
GentleBoost	88.85	316.14	2.31	222.89
LogitBoost	88.69	281.94	1.51	176.29

**Table 6:** Comparison of RMSE and R^2^ score between boosting and other ML models.

Model	Author	RMSE (dB)	R^2^ Score
MLR	González-Palacio et al. [1]	1.8401	0.9177
ANN	González-Palacio et al. [30]	1.613	0.9350
RF	González-Palacio et al. [30]	1.566	0.9389
SVR	González-Palacio et al. [30]	1.626	0.9342
AdaBoost	This study	0.5867	0.5261
XGBoost	This study	0.5190	0.7097
LGBM	This study	0.5166	0.7151
GentleBoost	This study	0.5778	0.5541
LogitBoost	This study	0.5820	0.5411

**Table 7:** Comparison of RMSE and R^2^ score between boosting and theoretical PL models.

Model	RMSE (dB)	R^2^ Score
AdaBoost	0.5867	0.5261
XGBoost	0.5190	0.7097
LGBM	0.5166	0.7151
GentleBoost	0.5778	0.5541
LogitBoost	0.5820	0.5411
Log-Distance	2.5645	0.8517
Okumura-Hata	32.1700	0.8054

## Data Availability

The data presented in this study are available in the GitHub repository at the following link: https://github.com/magonzalezudem/MDPI_LoRaWAN_Dataset_With_Environmental_Variables (accessed on 25 April 2025). These data were derived from publicly available resources.

## Abbreviations

The following abbreviations are used in this manuscript:

AdaBoost	Adaptive Boosting
ADR	Adaptive Data Rate
bp	Barometric Pressure
DL	Deep Learning
EFB	Exclusive Feature Bundling
EN	End Nodes
FSK	Frequency Shift Keying
GOSS	Gradient-Based One-Side Sampling
IoT	Internet of Things
ISM	Industrial, Scientific, and Medical
LightGBM	Light Gradient Boosting Machine
LogitBoost	Logistic Regression-based Boosting Algorithm
LoRaWAN	Long-Range Wide-Area Network
LoS	Line of Sight
LPWAN	Long-Range Low-Power Wide-Area Network
LSTM	Long Short-Term Memory
MAE	Mean Absolute Error
ML	Machine Learning
MLR	Multiple Linear Regression
MSE	Mean Squared Error
PL	Path Loss
PM	Particulate Matter
PSO	Particle Swarm Optimization
R^2^	Coefficient of Determination
rh	Relative Humidity
RMSE	Root Mean Squared Error
RSSI	Received Signal Strength Indicator
SNR	Signal-to-Noise Ratio
XGBoost	Extreme Gradient Boosting
